# Meibomian glands dropout in patients with inactive thyroid related orbitopathy

**DOI:** 10.1371/journal.pone.0250617

**Published:** 2021-04-22

**Authors:** Vannarut Satitpitakul, Tanavadee Rattanaphong, Vannakorn Pruksakorn

**Affiliations:** 1 Faculty of Medicine, Department of Ophthalmology, Center of Excellence for cornea and stem cell transplantation, Chulalongkorn University and King Chulalongkorn Memorial Hospital, Thai Red Cross Society, Bangkok, Thailand; 2 Faculty of Medicine, Department of Ophthalmology, Chulalongkorn University and King Chulalongkorn Memorial Hospital, Thai Red Cross Society, Bangkok, Thailand; Xiamen University, CHINA

## Abstract

**Purpose:**

To evaluate the structure and function of meibomian glands in patients with thyroid related orbitopathy (TRO) compared with age- and sex-matched controls without TRO.

**Methods:**

This cross-sectional study included 106 eyes of 53 patients with TRO and 106 eyes of 53 age- and sex-matched controls without TRO. Patients with TRO were assessed for thyroid hormone status, activity and severity of TRO. All participants completed OSDI questionnaires. Their meibomian glands’ structure and function were assessed, including the area of meibomian gland dropout, lipid layer thickness (LLT), meibum expressibility and quality scores, tear break-up time (TBUT), corneal and conjunctival staining scores. A generalized estimating equation (GEE) was used to compare between the two groups. The correlations between the area of meibomian gland dropout with symptoms and signs of TRO were evaluated using GEE and Spearman correlation.

**Results:**

All patients with TRO had inactive status. The mean area of meibomian gland dropout was higher in the TRO group (34.5±11.2%) compared with that of controls (30.1±10.7%, P = 0.03). Both mean meibum quality (TRO, 1.6±0.7; Controls, 2.0 ±0.5) and expressibility (TRO, 1.5 ±0.7; Controls, 1.7 ±0.6) scores were slightly better in the TRO group compared with those of controls (P = 0.01). There was no significant difference in OSDI, corneal and conjunctival staining, TBUT and LLT. The area of meibomian gland dropout in patients with TRO was correlated with euthyroid status (P<0.05) and lagophthalmos (P = 0.03).

**Conclusions:**

Patients with inactive TRO showed significantly higher meibomian gland dropout compared with that of age- and sex-matched controls without TRO.

## Introduction

Thyroid related orbitopathy (TRO) is an orbital inflammatory disorder characterized by changes in the periocular soft tissue. Various clinical findings can be presented in patients with TRO: proptosis, lid lag, lid retraction, lagophthalmos, strabismus and, in severe cases, optic neuropathy and corneal breakdown [1]. A significant number of patients with TRO have been suffered from ocular surface problems. Previous studies have reported the ocular surface changes in TRO associated with aqueous tear deficiency, proptosis, eyelid retraction and lagophthalmos, leading to dry eye disease in patients with TRO [2–4]. However, only a few studies evaluated the association of TRO with meibomian glands.

Meibomian gland dysfunction (MGD), a common multifactorial chronic ocular surface problem, is known to be the leading cause of dry eye disease affecting millions of people around the world. MGD is characterized by terminal duct obstruction and/or change in the glandular secretion, leading to the alteration of meibum secretion, meibum expression, meibomian gland dropout and deficiency of tear film lipid layer [5]. Patients suffering from TRO have been demonstrated to have a higher prevalence of obstructive MGD compared with that of normal controls [6]. In this study, we aimed to evaluate both structure and function of meibomian glands in patients with TRO compared with age- and sex-match control without TRO.

## Materials and methods

This prospective observational case-controlled study included 106 eyes of 53 patients with TRO and 106 eyes of 53 age- and sex-matched controls with no history of TRO from an outpatient clinic, King Chulalongkorn Memorial Hospital, Bangkok, Thailand, from January 2018 to July 2019. Ethical approval was obtained from the Institutional Review Board, Faculty of Medicine, Chulalongkorn University, Thailand, and the study adhered to the tenets of the Declaration of Helsinki. Written informed consent forms were obtained from all participants before enrollment.

TRO patients aged between 18 to 80 years were enrolled. The diagnosis of TRO was made when 2 of the following 3 signs were presented; 1) concurrent or recently treated immune-related thyroid dysfunction, including Graves’ hyperthyroidism, Hashimoto thyroiditis and circulating thyroid antibodies, 2) any signs and symptoms that involve eyelid retraction, fluctuating eyelid edema or erythematous, chemosis, caruncular edema, proptosis, restrictive strabismus and compressive optic neuropathy, 3) fusiform enlargement of extraocular muscles by computer tomography assessment [7]. Patients were excluded if they were regular contact lens wearers, glaucoma medication users, or had a history of or concurrent MGD treatments. The 1 to 1 age- and sex-matched control participants were randomly recruited from hospital personnel. Exclusion criteria for controls were history or any signs of either thyroid diseases or TRO, and history of or concurrent MGD treatments.

All participants provided demographic data including age, sex and history of ophthalmic and systemic diseases. In the TRO group, patients were also evaluated for thyroid hormone status, previous or concurrent treatments of thyroid disease, activity, and severity of TRO. The activity of TRO was assessed using a clinical activity score (CAS) [8]. CAS, evaluated based on the classical features of inflammation in TRO, was the sum of the following 7 items presented: spontaneous retrobulbar pain, pain on attempted up- or down gaze, redness of the eyelids, redness of conjunctiva, swelling of the eyelids, inflammation of the caruncle and/or plica and conjunctival edema. CAS less than 3 out of 7 was considered as inactive. The severity of TRO was classified into mild, moderate to severe and sight threatening. The severity was classified as mild in the case that the patients had one or more of the following conditions: lid retraction less than 2 mm, mild soft tissue involvement, exophthalmos less than 3 mm, transient or no diplopia and corneal exposure responsive to lubricants. It was defined as moderate to severe when patients had any one or more of the following conditions: lid retraction at least 2 mm, moderate or severe soft tissue involvement, exophthalmos at least 3 mm and diplopia. Lastly, it was specified as the sight-threatening TRO when patients had dysthyroid optic neuropathy and/ or corneal breakdown [8].

Consequently, all participants’ eyes were evaluated by ophthalmic examinations as follows: ocular surface disease index (OSDI) questionnaire, best corrected visual acuity, lipid layer thickness of tear films using tear interferometer (LipiView^®^, TearScience Inc, NC, USA), meibograph of the upper eyelid (Oculus^®^ Keratograph 5M, Oculus^®^, WA, USA), tear film break-up time (TBUT), conjunctival and corneal staining scores according to National Eye Institute (NEI), meibum quality, meibum expressibility, measurement of proptosis using Hertel exophthalmometer and measurement of lagophthalmos.

The area of meibomian gland dropout was analyzed from meibography using ImageJ software. To do this, a meibograph image was exported and then analyzed. First, the area covered by meibomian glands and the area of tarsal conjunctiva were traced using a freehand tool and measured in μm^2^ (S1 Fig). Second, the area of meibomian gland dropout was calculated by the total area of tarsal conjunctiva minus area covered by meibomian glands and then presented as a percentage of the total area of tarsal conjunctiva. Two independent masked observers performed all measurements, and the average values were used for the analysis. Meibomian glands were examined under a slit lamp biomicroscopy. Meibum quality was assessed in eight glands of central lower eyelids using the Meibomian Gland Evaluator (TearScience Inc, NC, USA). Meibum quality was graded on a scale as follows: 0, clear; 1, cloudy; 2, cloudy with debris; and 3, solid paste or no expression. For meibum expressibility, five central meibomian glands of the lower eyelid were evaluated using the Meibomian Gland Evaluator and graded based on a number of glands that can be expressed as follows: 0, all glands; 1, 3–4 glands; 2, 1–2 glands; 3, no gland.

Areas of meibomian gland dropout, lipid layer thickness, OSDI scores, conjunctival and corneal staining scores, TBUT, meibum quality and meibum expressibility scores were compared between the TRO group and the control group using a generalized estimating equation. Furthermore, among the TRO group, the difference between the area of meibomian gland dropout among patients with hypothyroidism, hyperthyroidism and euthyroidism was evaluated using a generalized estimating equation. A generalized estimating equation was used to compare between groups and accounted for the correlated nature of paired eye outcomes [9]. The correlation between CAS, lagophthalmos and proptosis with areas of meibomian gland dropout were also evaluated using spearman correlation. All statistical analysis was performed using Stata Statistical Software: Release 14. (Collage Station, TX, StataCorp LP.) P value less than 0.05 was considered significant.

## Results and discussion

This study included 106 eyes of 53 participants (37 females and 16 males) with a mean age of 51.2±14.6 years (range, 22–77 years) in the TRO group and 106 eyes of 53 participants (37 females and 16 males) with a mean age of 51.3±15.8 years (range, 22–78 years) in the control group. There was no significant difference in age and sex between those two groups (P = 1.00 and 0.97). The mean lagophthalmos were 0.5±1.0 mm in the TRO group and 0.1±0.4 mm in the control group (P = 0.01) and the mean proptosis were 18±3 mm in the TRO group and 13±2 mm in the control group (P<0.0001). Ophthalmic and systemic diseases in each group were shown in Table 1.

**Table 1:** The ophthalmic and systemic diseases in thyroid related orbitopathy (TRO) and control groups.

	TRO group (N = 106 eyes of 53 participants)	Control group (N = 106 eyes of 53 participants)
Ophthalmic diseases, eye (%)		
Pterygium	1 (0.9)	2 (1.9)
Strabismus	2 (1.9)	0
Diabetes retinopathy	0	1 (0.9)
Systemic diseases, participants (%)		
Dyslipidemia	14 (26.4)	14 (26.4)
Hypertension	18 (34.0)	14 (26.4)
Diabetes mellitus	12 (22.6)	8 (15.1)
Menopause	18 (34.0)	18 (34.0)
Others	8 (15.1)	11 (20.8)

Among the TRO group, at the time of enrollment, 18 (34.0%), 30 (56.6%) and 5 (9.4%) participants had hypothyroid, hyperthyroid and euthyroid status, respectively. Twenty-eight (52.8%) participants had mild TRO and 25 (47.2%) participants had moderate to severe TRO. All participants had inactive TRO status.

The mean areas of meibomian gland dropout were 34.5±11.2% in the TRO group and 30.1± 10.7% in the control group. The area of meibomian gland dropout was significantly higher in the TRO group compared with that of the control group (P = 0.03). The mean lipid layer thicknesses were 68.2±24.6 ICU in the TRO group and 62.3±27.2 ICU in the control group. There was no significant difference in lipid layer thickness between the two groups (P = 0.20).

The OSDI scores, conjunctival staining scores, corneal staining scores, TBUT, meibum quality and meibum expressibility scores of TRO and control groups were shown in Table 2. Both meibum quality and meibum expressibility scores were significantly lower in TRO groups compared with those of controls. There was no difference in OSDI scores, conjunctival staining scores, corneal staining scores and TBUT between the groups.

**Table 2:** Ocular surface symptoms and signs in thyroid related orbitopathy (TRO) and control groups.

	TRO group (N = 106 eyes)	Control group (N = 106 eyes)	P value^a^
OSDI scores (mean ±SD)	20.3 ±17.3	24.7 ±18.3	0.20
Conjunctival staining scores (NEI; mean ±SD)	0.4 ±0.8	0.3 ±0.6	0.90
Corneal staining scores (NEI; mean ±SD)	1.4 ±1.7	1.3 ±2.2	0.33
Tear film break-up time (seconds; mean ±SD)	4 ±2	4 ±2	0.37
Meibum quality scores (mean ±SD)	1.6 ±0.7	2.0 ±0.5	0.01
Meibum expressibility scores (mean ±SD)	1.5 ±0.7	1.7 ±0.6	0.01

^a^Generalized estimating equation.

Among the TRO group, the mean areas of meibomian gland dropout in patients with hypothyroidism, hyperthyroidism and euthyroidism were 30.6±10.9, 36.0±7.9 and 48.9±11.9, respectively. Patients with euthyroidism showed significantly higher meibomian gland dropout compared with the meibomian gland dropout of patients with hypothyroidism (P = 0.046) and hyperthyroidism (P = 0.01). There was no significant difference in the area of meibomian gland dropout between patients with hypothyroidism and hyperthyroidism (P = 0.08). In addition, the area of meibomian gland dropout was correlated with lagophthalmos (r = 0.21, P = 0.03). There was no correlation between the area of meibomian gland dropout with CAS (r = -0.05, P = 0.70) and proptosis (r = 0.02, P = 0.88).

Patients with TRO showed significantly higher meibomian gland dropout but lower meibum quality and meibum expressibility scores compared with the scores of age- and sex-matched controls. Our study demonstrated that meibomian gland dropout in patients with TRO correlated with thyroid hormone status and lagophthalmos. Patients with euthyroidism showed more advanced meibomian gland dropout compared with the meibomian gland dropout of patients with hypo- and hyperthyroidism.

Several studies have reported the ocular surface changes leading to dry eye disease in patients with TRO [2–4,10]. However, a few studies have focused on the structure and function of meibomian glands. In this study, we calculated and compared the exact areas of meibomian gland dropout in patients with inactive TRO with those of age- and sex-matched participants without TRO, using ImageJ software. We found that the area of meibomian gland loss was higher in patients with inactive TRO. Although meibomian gland dropout in TRO has previously been reported [10], the significant meibomian gland dropout in patients with inactive TRO has never been studied. The preceding study of Park J [10] and Wong CY [11] included both active and inactive TRO and graded the meibomian gland dropout using meiboscore to specify a scale of meibomian gland loss ranges from 0 to 6. In contrast, our study calculated the exact areas of meibomian gland loss by two independent masked observers using Image J software. Park J [10] research demonstrated higher meibomian gland dropout in patients with both active and inactive TRO compared with that of normal controls. The studies also reported that patients with active TRO had significantly higher meibomian gland dropout compared with that of patients with inactive TRO [10,11]. In addition, they further presented the positive correlation between the CAS scores and the degree of meibomian gland dropout among patients with active and inactive TRO. Interestingly, according to our study, the correlation between the CAS scores and the area of meibomian gland dropout was not found in patients with inactive TRO.

The exact mechanism of meibomian gland dropout in patients with TRO is still unclear. The possible mechanism has been proposed to be due to ocular surface inflammation associated with the inflammation within the orbit [10]. Ocular surface inflammation leading to the alteration of both structure and function of meibomian glands has previously been described in various conditions including conjunctivitis, contact lens wearing and Stevens-Johnson syndrome [12]. In this study, we found that patients with TRO with none or minimal inflammatory activity of TRO detected by CAS, a widely accepted score, could still lead to significant meibomian gland loss compared with that of patients without TRO. Therefore, we thought that even without clinically detected ocular inflammatory activity, patients with inactive TRO had a certain level of ocular surface inflammation. Over the past years, the higher meibomian gland dropout has also been demonstrated in patients with Hashimoto’s thyroiditis without TRO, compared with that of healthy controls [13]. Considering the potential destruction of the meibomian gland in patients with inactive TRO, early detection and management of meibomian gland loss is required. Such objective measures may be used in the future for evaluating the inflammatory activity in patients with TRO at subclinical levels. Further studies evaluating the potential of anti-inflammatory medications for preventing meibomian gland loss in patients with TRO is also needed.

Despite higher meibomian gland loss in patients with TRO, there was no significant difference in LLT between patients with TRO and without TRO in our study. This may imply that the remaining meibomian glands of patients with TRO are still sufficient to secrete meibum into the tear film. The previous study on the treatment response in patients with meibomian gland dysfunction has shown that even in patients with more than 40% of meibomian gland dropout, the remaining meibomian glands were able to maintain a sufficient lipid layer of the tear film [14]. However, the threshold beyond which remaining meibomian gland is not adequate to maintain the lipid layer is still uncertain.

While most studies reported none or weak correlation between dry eye symptoms and signs [15,16], a significant correlation between LLT and dry eye symptoms had been described [17–19]. The maintenance of LLT would partially explain the similar degrees of dry eye symptoms according to OSDI scores in our study. Moreover, with no difference in LLT, we also found that TBUT, conjunctival and corneal staining scores showed no significant difference.

Interestingly, we found that meibum quality and expressibility scores were slightly better in patients with TRO. Despite the increased palpebral width in patients with TRO that potentially leads to the damage of the ocular surface, better meibum quality and expressibility scores were presented. This would explain the result of no difference in dry eye manifestations and LLT, between patients with and without TRO in our study. In contrast to our results, Park J [10] showed no difference in meibum quality and meibum expressibility scores in patients with both active and inactive TRO compared with the scores of patients without TRO. We believed that the activity of TRO might be a factor involving both the quality and expressibility of meibomian gland. A future study about the reasons for such varying results in meibum quality and expressibility in patients with TRO is yet to be figured out. We hypothesized that the better meibum quality and expressibility found in our study could in part be related to the forceful blinking due to wider palpebral fissures in patients with TRO and the compensatory mechanism of meibomian glands in patients with gland dropout when the remaining meibomian glands were adequate. Thus, patients with TRO, who had a widening palpebral fissure, were initially able to maintain the lipid layer of the tear film and consequently delayed the destruction of the ocular surface.

In our study, patients with TRO with euthyroidism demonstrated significantly more advanced meibomian gland dropout compared with that of patients with hypo- and hyperthyroidism. This result was in contrast with the previous report on the severity of TRO, which demonstrated more severe TRO in patients with hypo- and hyperthyroidism when considering the incidence of surgical treatments reflected the severity of disease [20].

There were some limitations in this study. First, this was a cross-sectional study. Therefore, various natural histories of thyroid diseases may be enrolled in the TRO group. In addition, factors that might be associated with meibomian gland dropout such as duration of thyroid disease, duration of TRO, medication and intervention received were not evaluated in this study.

## Conclusions

We demonstrated the significant meibomian gland dropout in patients with inactive TRO compared with that of age- and sex-matched controls. The slightly better meibum quality and expressibility scores in the TRO group along with no difference in LLT were found. The higher area of meibomian gland loss in patients with TRO was correlated with euthyroid status and lagophthalmos. On the basis of these findings, we suggest that ophthalmologists should examine meibomian glands’ structure, in addition to their function, for early detection, management and prevention of further meibomian gland destruction in patients with inactive TRO.

## Supporting information

S1 Fig Method for determining the meibomian gland dropout. The Polygonal Selection tool of ImageJ was used to determine the area of meibomian glands (A) and tarsal conjunctiva (B).
(TIF)
