# Supplementary figures and images for: Meibomian glands dropout in patients with inactive thyroid related orbitopathy
Source: PLoS One. 2021 Apr 22;16(4):e0250617. doi: 10.1371/journal.pone.0250617 (PMC8061908; doi:10.1371/journal.pone.0250617)

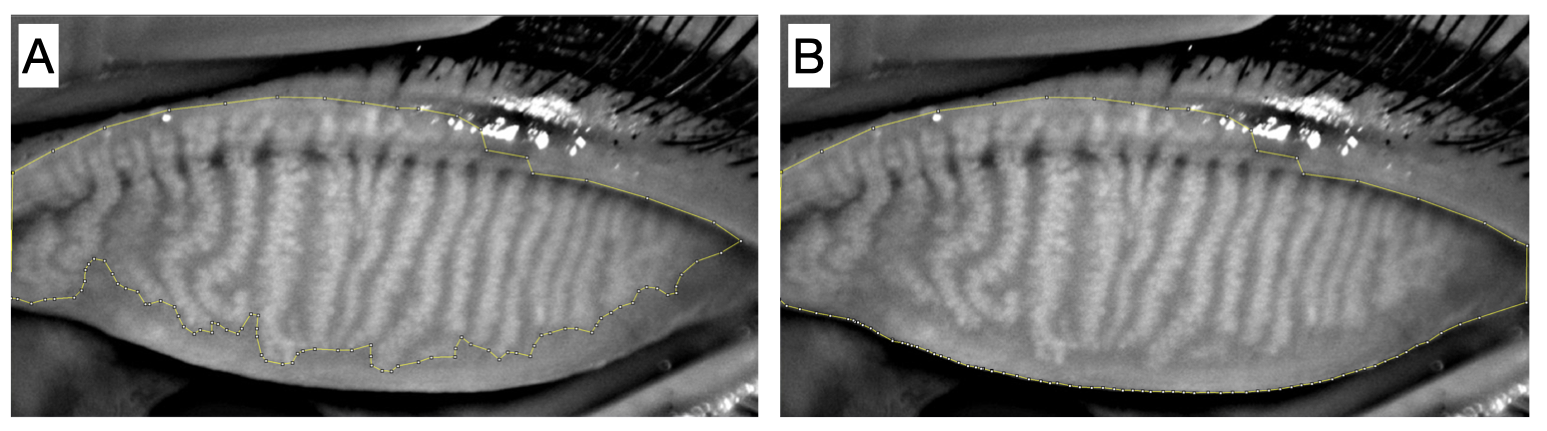

Supplement: S1 Fig — The Polygonal Selection tool of ImageJ was used to determine the area of meibomian glands (A) and tarsal conjunctiva (B). (TIF) [file pone.0250617.s001.tif]
